# Measuring the effect of deprivation on primary health care performance using data envelopment analysis and Malmquist Indices

**DOI:** 10.1007/s10729-025-09715-9

**Published:** 2025-06-07

**Authors:** Holly Bea Merelie, Carla Alexandra Filipe Amado, Sérgio Pereira dos Santos

**Affiliations:** Faculty of Economics and Center for Advanced Studies in Management and Economics (CEFAGE), University of Algarve, Faro, Portugal

**Keywords:** Data envelopment analysis, Malmquist index, Primary health care, Deprivation

## Abstract

Life expectancy is typically shorter in areas with higher deprivation, highlighting the need for policymakers and health care managers to focus on reducing health inequalities through efficient and effective care. This study aims to assess the impact of deprivation on primary health care performance using data from the National Health Service (NHS) in England. Two methods are applied: Data Envelopment Analysis (DEA) to evaluate the performance of 188 Clinical Commissioning Groups (CCGs), whose duties were recently taken on by the new Integrated Care Systems (ICSs), and the Malmquist Index (MI) to assess deprivation’s effect on performance. The DEA results reveal significant variation among CCGs in equity, efficiency, and effectiveness, indicating substantial room for improvement. The MI results show that while CCGs in more deprived areas had more resources per capita and higher efficiency, they were generally less effective than those in less deprived areas. This emphasizes the need to enhance health and social policies to address persistent health inequalities due to deprivation, a critical challenge for the new ICSs. This study illustrates how DEA and the MI can support policymakers and managers in this effort.

## Highlights

- Deprivation has a negative impact on life expectancy.
- Data Envelopment Analysis and the Malmquist Index are applied to evaluate the impact of deprivation on primary health care performance.
- Data from 188 Clinical Commissioning Groups (CCGs) for the calendar year 2019 is used.
- The results show that primary care providers in more deprived areas had more resources per capita and higher efficiency but were overall less effective compared to those in less deprived areas.
- The findings highlight the importance of strengthening existing health and social policies to effectively address the persistent health inequalities caused by deprivation.

## Introduction

Health inequalities can be defined as avoidable and unfair differences in health between groups of people and involve health status, access to care, quality and experience of care, behavioural risks to health and wider determinants of health [1]. Although addressing inequality in health care has long been considered a priority [2], a study carried out by the Organisation for Economic Co-operation and Development (OECD) found that disadvantaged groups exhibit poorer health behaviours, lower health education, and reduced access to doctors and preventive services, with unmet health needs concentrated among low-income populations [3].

In England, the focus of this research, the Index of Multiple Deprivation (IMD) is the official measure of relative disadvantage, providing a comprehensive assessment of deprivation across various domains, including income, employment, education, health, crime, housing, and living environment [4]. The more deprived an area is, the higher the IMD score.

Data from 2017–2019 show that there is a systematic relationship between deprivation and life expectancy (LE), existing a gap in LE of almost 8 years between women who live in the 10% least-deprived areas (higher LE) compared to those in the 10% most-deprived areas (lower LE) [1]. This gap is even wider among men – about 9.4 years between the least and most deprived areas [1].

Primary health care (PHC) has long been considered crucial for addressing health inequalities as it is the first point of contact for individuals and communities with the national health system, providing care close to where people live and work [2]. However, PHC systems face challenges such as an aging population with complex health needs, increased consultation complexity, poor patient satisfaction, workforce recruitment and retention issues, and limited resources [5]. Therefore, it is essential for PHC providers to deliver equitable, efficient and effective care.

In England, the planning and purchasing of NHS services targeted at ensuring equitable, effective, and efficient PHC services used to be carried out by Clinical Commissioning Groups (CCGs), which were recently replaced by Integrated Care Systems (ICSs). Each ICS has an Integrated Care Board (ICB), which are NHS organizations responsible for planning health services in their ICS area for their local population, replacing, in this way, the functions of the CCGs. Considering that the CCGs responsibilities, staff, and resources were transferred to the ICBs, which in some cases cover the same catchment areas, our findings remain relevant.

While excelling in the three performance dimensions above is paramount for any health organization, measuring the equity, efficiency and effectiveness of the services delivered is not an easy undertaking with some authors identifying PHC as a complex setting to measure performance (e.g. [5]). In spite of this, previous studies have shown that Data Envelopment Analysis (DEA) can be a valuable tool to measure performance in this context. For recent systematic reviews on the use of DEA to measure performance in primary care, the reader is referred to Zakowska and Godycki-Cwirko [6] and Pelone et al. [7].

Data Envelopment Analysis, developed by Charnes et al. [8], is a non-parametric programming technique that was developed to measure efficiency of not-for-profit entities in public programmes [9]. DEA measures relative efficiency of homogenous Decision-Making Units (DMUs) by employing mathematical programming to control and evaluate past accomplishments and to aid in planning future activities [9]. A recent systematic review carried out by Neri et al. [5] shows that DEA is the most frequently utilized approach to measure performance in PHC.

The second technique used in this research is the Malmquist Index (MI), first developed by Fare et al. [10], but in the version proposed by Camanho and Dyson [11]. According to this latter version, the DEA technique and the MI can be used not to measure productivity changes over time, but rather to perform a cross-sectional performance comparison of groups of DMUs operating in different conditions at one specific moment in time. The use of this adapted version of the MI is advantageous as it allows us to compare the performance of different groups of CCGs based on their level of deprivation, making a significant contribution to the literature. This comparison is made in terms of both the best practice frontier and the distance to the best practice frontier. While the traditional approach would evaluate the productivity change of a sample of CCGs between two time periods without accounting for deprivation levels, the modified version makes the impact of deprivation visible by contrasting two groups of CCGs within a single period.

Considering that we aim to assess the performance of the CCGs operating in England until 2022, as well as the impact of deprivation on their performance, to derive learning for the newly formed ICSs, the use of an approach combining the two techniques above is well-suited to achieve these objectives. Specifically, DEA will have a two-fold purpose. Firstly, it will allow us to identify CCGs that demonstrated resource equity, service efficiency and service effectiveness, and to explore if CCGs operating in areas with different deprivation levels displayed differences in the performance results obtained. Secondly, it will allow us to identify benchmarks that may be used to improve the performance of the new ICSs operating in the same areas of the underperforming CCGs and that have taken on their duties. In turn, the MI will allow us to quantify the effect of deprivation on CCGs’ performance in terms of resource equity, service efficiency and service effectiveness. Ultimately, these objectives will offer valuable information to enhance the understanding of the impact of deprivation on the performance of PHC units. In this context, our study provides specific insights into how deprivation influences primary health care performance within the NHS in England.

These insights can be directly linked to ongoing NHS strategies aimed at addressing health inequalities, such as those outlined in the NHS Long Term Plan in 2019 [12] and the National Healthcare Inequalities Improvement Programme in 2021 [13]. Considering that one of the most important aims of ICSs is to reduce health inequalities, examining the impact of deprivation on primary care delivery is of paramount importance. Additionally, analysing several performance criteria -equity, efficiency and effectiveness -helps policymakers and managers focus their efforts on areas with the greatest potential for improvement. Lastly, identifying performance benchmarks for each of these criteria is crucial for understanding which structures and processes work best in different areas.

To achieve the aims set out previously, the remainder of this article is organized as follows: Sect. 2 provides an overall description of the CCGs and highlights some health inequalities that are present, followed by a literature review of relevant studies that have applied DEA and the MI to PHC; Sect. 3 presents the DEA and the MI models used in the empirical analysis, discusses the main results obtained and explores their practical and policy implications; finally, Sect. 4, concludes the article by highlighting the key messages from the research, limitations and suggestions for further research.

## Literature review

### Clinical commissioning groups and health inequalities

In the United Kingdom (UK), Clinical Commissioning Groups were established as part of the Health and Social Care Act in 2012 replacing Primary Care Trusts (PCT) and were in operation until July 2022. The main duty of these groups was to commission, which is a continual process of planning, agreeing, and monitoring services, ranging from assessing health-needs, to designing clinical pathways for patients, defining service specifications, and developing contract negotiation or procurement, while continuously assessing quality [14].

Considering that PHC offers the first point of access to the health care system, CCGs, and now the ICSs, have an increased responsibility for delivering equitable, efficient, and effective care. In view of this, a proactive effort to improve performance within PHC in the NHS is needed to certify that the government’s investment meets the needs of a growing population with constantly more complex and expensive health care needs [5]. In addition, it is fundamental that health inequalities, which may arise due to the conditions in which we are born, grow up, live, work, and age, are properly addressed, as these conditions affect not only the opportunities for good health but also influence our mental health, physical health, and overall well-being [1].

Although it is common to examine inequality in terms of income and socio-economic factors, other factors such as disability, sex, and ethnicity have also been shown to generate health inequalities (e.g., [12, 13, 15]). Due to these findings, the NHS Long Term Plan in 2019, concentrated efforts on reducing health inequalities and unwarranted variations in care. To achieve this, the NHS set out strategies such as the allocation of a higher share of funding towards geographies with high health inequalities. Specific and measurable goals were also set for narrowing inequalities related with screening and vaccination programmes, pregnancy, labour and postnatal care and mental health, among others 12.

### Data envelopment analysis applied to primary health care

Farrell [16] was the first author to develop an empirical method to calculate relative productive efficiency of a set of DMUs. This method was further developed by Charnes et al. [8] who named it by Data Envelopment Analysis. DEA is a non-parametric programming technique that was developed to measure efficiency of non-for-profit entities in public programmes.

In the Constant Returns to Scale (CRS) model, also known as the CCR model, the efficiency rate of each DMU is determined by the maximum ratio between the weighted sum of outputs to the weighted sum of inputs subject to the condition that similar ratios for every DMU cannot exceed unity. The envelopment form of the linear programme for assessing the technical efficiency of a DMU with output orientation under a CRS technology can be represented by:

$${E}_{0}=\text{Max }{h}_{j0}+\varepsilon \left[\sum_{i=1}^{m}{I}_{i}+\sum_{r=1}^{s}{O}_{r}\right]$$

Subject to

$$\begin{array}{cc}\sum_{j=1}^{N}{\alpha }_{j}{x}_{ij}={x}_{i{j}_{0}}-{I}_{i }& \text{i}=1...\text{m}\end{array}$$

$$\begin{array}{cc}\sum_{j=1}^{N}{\alpha }_{j}{y}_{rj}={O}_{r}+{h}_{j0} {y}_{{rj}_{0}}& \text{r}=1...\text{s}\end{array}$$

$${\alpha }_{j}\ge 0, j=1\dots N, {I}_{i}, {O}_{r}\ge 0\;\forall\;i\; \text{and}\;r, {h}_{j0}\;\text{free}$$

$\varepsilon$ is a non-Archimedean infinitesimal.

Where $N$ DMUs (j = 1… $N$) use $m$ inputs to secure $s$ outputs. The model gives priority to maximizing ${h}_{j0}$, by identifying a point which offers output levels reflecting the maximum feasible radial expansion of the output levels without raising the input levels [17]. The variables ${I}_{i}$ and ${O}_{r}$ are slack variables that represent additional feasible input reductions and/or output augmentations beyond the radial expansion of the output levels of DMU ${j}_{0}$ [17].

In turn, the linear programme for assessing technical efficiency with output orientation under a Variable Returns to Scale (VRS) technology can be represented by:

$${E}_{0}=\text{Max }z+\varepsilon \left[\sum_{i=1}^{m}{I}_{i}+ \sum_{r=1}^{s}{O}_{r}\right]$$

Subject to

$$\begin{array}{cc}{\sum }_{j=1}^{N}{\alpha }_{j}{x}_{ij}={x}_{i{j}_{0}}-{I}_{i}& \text{i}=1\dots \text{m}\end{array}$$

$$\begin{array}{cc}{\sum }_{j=1}^{N}{\alpha }_{j}{y}_{rj}={O}_{r}+{z y}_{{rj}_{0}}& \text{r}=1...\text{s}\end{array}$$

$${\sum }_{j=1}^{N}{\alpha }_{j}=1$$

$${\alpha }_{j}\ge 0, j=1\dots N, {I}_{i}, {O}_{r}\ge 0\;\forall\;i\;\text{and}\;r, z\;\text{free}.$$

From the envelopment linear programming models presented above, it is possible to formulate the multiplier version of these models to calculate the input and output weights.

Although the DEA technique presents some pitfalls [18], it has been vastly applied in the health care setting. In fact, recent systematic reviews by Neri et al. [5] and Zakowska and Godycki-Cwirko [6] concluded that DEA is the most commonly used method for assessing efficiency in primary health care.

The studies using DEA to assess performance in PHC have covered several countries, including Brazil [19], China [20, 21], Czech Republic [22], Greece [23], Mexico [24], Portugal [25, 26], Spain [27], Turkey [28], and the UK [29–32]. Although all these studies focus on PHC, different types of DMUs were analysed including regions, health centres, district health authorities (DHA) and CCGs.

The most common objective of the studies carried out so far, has been to use DEA to measure multiple dimensions of performance including efficiency, effectiveness and equity [23–26, 28, 30–33]. Others, have applied DEA to measure the efficiency of health care resource allocation [22, 34, 35]. While still others have used DEA to assess the impact of several factors on the performance of PHC providers [25, 27, 33].

So far, to the best of the authors’ knowledge, only two studies have employed DEA to assess the performance of CCGs. The first study was carried out by Takundwa et al. [30], who identified 47 of the 208 CCGs analysed as efficient, with the inefficient units showing an average technical efficiency score of 90%. These authors also demonstrated that three environmental factors were significant predictors of efficiency: CCGs with smaller population sizes were more efficient and high unemployment and high prevalence of Chronic Obstructive Pulmonary Disease (COPD) decreased efficiency. Deprivation was not identified as a significant predictor of efficiency. The second study was carried out by Williams et al. [29], and assesses the relative productivity of primary medical services in England and the impact of the COVID-19 pandemic on their productivity levels using data for 101 CCGs. Interestingly, their study reveals minimal geographic differences in the productivity of primary medical services when assessed at the clinical commissioning group level and a slight decline in productivity during the pandemic.

### Malmquist Indices applied to primary health care

The Malmquist Index (MI) was introduced by Caves et al. [36] and further developed for performance assessment by Färe et al. [10]. However, the Malmquist Productivity Index (MPI) developed by Färe et al. [10] measures productivity change over time. Therefore, in this research, we use the adaptation of the MI proposed by Camanho and Dyson [11], which no longer measures productivity change over time but allows a cross-sectional performance comparison of groups of DMUs operating in different conditions at one moment in time.

This method allows the decomposition of the new performance index (Eq. 1) into two indices: one that reflects efficiency spread among DMUs operating in similar conditions and another that reflects the performance gap between the best-practice frontiers of the different groups (Eq. 2) as shown in the formulas that follow:

1 $${I}^{AB}={\left[\frac{{\left({\prod }_{j=1}^{\delta A}{E}^{A}\left({X}_{j}^{A}.{Y}_{j}^{A}\right)\right)}^{\frac{1}{\delta A}}}{{\left({\prod }_{j=1}^{\delta B}{E}^{A}\left({X}_{j}^{B}.{Y}_{j}^{B}\right)\right)}^{\frac{1}{\delta B}}} . \frac{{\left({\prod }_{j=1}^{\delta A}{E}^{B}\left({X}_{j}^{A}.{Y}_{j}^{A}\right)\right)}^{\frac{1}{\delta A}}}{{\left({\prod }_{j=1}^{\delta B}{E}^{B}\left({X}_{j}^{B}.{Y}_{j}^{B}\right)\right)}^{\frac{1}{\delta B}}} \right]}^\frac{1}{2}$$

The two ratios within the square brackets assess the performance of the DMUs with respect to a single reference technology. The first ratio measures the average performance of DMUs from group A, relative to group A’s frontier, and divides this by the average performance of DMUs from group B. The second ratio applies the same principle but relative to group B’s frontier. In consistency with the recommendation of Cooper et al. [37], when calculating the performance of the DMUs of a group with reference to the technology of a different group, the super-efficiency model, proposed by Andersen and Peterson [38], should be used allowing the measures to assume values above one.

The overall performance measure presented previously can be decomposed into two sub-components, as follows:

2 $${I}^{AB}= \frac{{\left[{\prod }_{j=1}^{\delta A}{E}^{A}\left({X}_{j}^{A}.{Y}_{j}^{A}\right)\right]}^{\frac{1}{\delta A}}}{{\left[{\prod }_{j=1}^{\delta B}{E}^{B}\left({X}_{j}^{B}.{Y}_{j}^{B}\right)\right]}^{\frac{1}{\delta B}}} . {\left[\frac{{\left({\prod }_{j=1}^{\delta A}{E}^{B}\left({X}_{j}^{A}.{Y}_{j}^{A}\right)\right)}^{\frac{1}{\delta A}}}{{\left({\prod }_{j=1}^{\delta A}{E}^{A}\left({X}_{j}^{A}.{Y}_{j}^{A}\right)\right)}^{\frac{1}{\delta A}}} . \frac{{\left({\prod }_{j=1}^{\delta B}{E}^{B}\left({X}_{j}^{B}.{Y}_{j}^{B}\right)\right)}^{\frac{1}{\delta B}}}{{\left({\prod }_{j=1}^{\delta B}{E}^{A}\left({X}_{j}^{B}.{Y}_{j}^{B}\right)\right)}^{\frac{1}{\delta B}}} \right]}^\frac{1}{2}$$

where the ratio outside the square brackets ($${IE}^{AB})$$ compares within-group performance spread and the ratio inside the square brackets ($${IF}^{AB})$$ evaluates the performance gap between the group frontiers [11]. The former reflects internal performance, whereas the latter reflects the context in which the DMUs are required to operate [11]. Thus, better performance is associated to less dispersion in performance levels within the group and/or dominance of the best practice frontier.

A value of ${IE}^{AB}$ less than one reflects that the efficiency spread is greater in the DMUs in group A than in those of group B, in other words, there is lower consistency in efficiency levels within group A compared to group B. A value of ${IF}^{AB}$ less than one reflects lower productivity of the frontier of group A compared to group B.

To the best of our knowledge, there are no published studies that have used this method to contrast the performance of primary health care providers belonging to groups with different characteristics. This explains why no applications of this method are discussed in this section.

In conclusion, while remarkable progress has been made in this area of research, it is commonly agreed that further research is still required. For example, Zakowska and Godycki-Cwirko [6] recognize that appropriate input and output variables and suitable DEA models for assessing PHC need to be researched further. Dlouhy [22] suggests that further research should be based on the use of DEA models for inequality measurement. Takundwa et al. [30] recommend that appropriate measures of output in health care should be used, and that guidelines for measuring efficiency in health care should be designed to promote more robust methodological processes and increased comparability across studies. Amado and Santos [26] alert for the need to account for the views of different stakeholders in DEA models. Amado and Dyson [31] suggest that variation in results, implicit trade-offs between performance dimensions and the impact of area deprivation are other issues that need further investigation. Cinaroglu [28] proposes the inclusion of welfare indicators in future efficiency analyses. Neri et al. [5] suggest that the impact of technological change and skill-mix on primary health care efficiency should be studied, and that future research should include more direct patient care staff, instead of focusing only on doctors and nurses.

This research aims to address several of these suggestions, offering a relevant contribution not only to theory but also to practice. Firstly, it seeks to compare the performance of CCGs. As previously mentioned, only two studies have been identified that use DEA to explore the performance of this type of DMUs. While both studies provide valuable insights, the study by Takundwa et al. [30] focuses exclusively on the efficiency of CCGs, and the study by Williams et al. [29] assesses the impact of the COVID-19 pandemic on the productivity levels of 101 CCGs, using the standard MPI to contrast their performance in two different periods. Our study expands on Takundwa et al. [30] by incorporating two additional dimensions of performance: equity and effectiveness. Our analysis also innovates beyond the study by Williams et al. [29] by including indicators of outcomes and using a modified version of the MPI not to compare the performance of units across two time periods, but rather to compare the performance of two groups of units facing significant differences in the levels of deprivation of the communities they serve. To the best of our knowledge, no previous studies have used this adapted version of the MPI to compare the performance of primary health care providers based on the level of deprivation.

Additionally, by developing models that include various categories of staff and incorporate both activity indicators and care outcomes, we address the recommendations of Neri et al. [5], Zakowska and Godycki-Cwirko [6], and Takundwa et al. [30], among others. Overall, the approach we propose will enable a more comprehensive assessment of the performance of CCGs and the likely impact of deprivation on their performance.

Although CCGs were replaced by ICSs in 2022, our findings clearly retain relevance. Firstly, because understanding how CCGs performed can provide a baseline for comparing the equity, efficiency and effectiveness of the new ICSs. Secondly, because many ICSs have taken on the duties of CCGs and therefore, understanding past performance helps in making informed decisions about resource allocation, ensuring that areas previously identified as underperforming receive the necessary support and attention. Thirdly, because analysing the impact of deprivation on the performance of CCGs can offer valuable lessons for ICSs, helping to avoid past mistakes and build on successful strategies. Lastly, because there is need for public accountability and transparency in how health services have been managed and delivered. Evaluating CCGs performance ensures that there is a comprehensive record of their equity, efficiency and effectiveness. Therefore, overall, our findings serve as a foundation for extracting insights into the challenges encountered by the NHS in enhancing population health and mitigating health disparities.

## Empirical analysis

For this research, DEA was the technique chosen to measure, in a first stage, the performance of each CCG, regarding equity of resource allocation, service efficiency and service effectiveness. Then, in a second stage, the CCGs were divided into two groups according to the IMD, and the MI proposed by Camanho and Dyson [11] was used to analyse group performance and identify health inequalities.

### The models used in the analysis

**Fig. 1:**
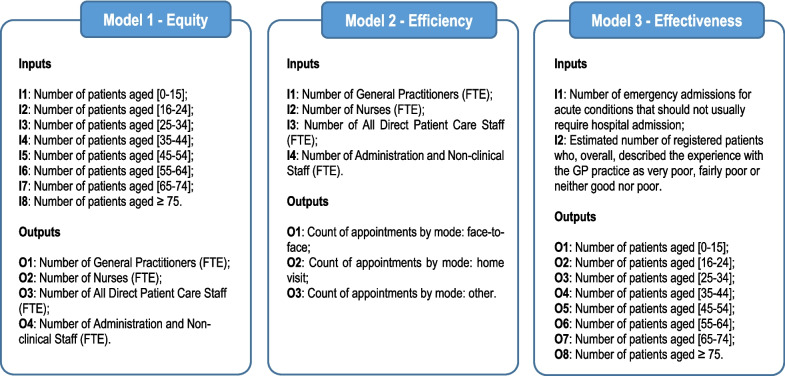
DEA Models used in the analysis

The choice of variables in this study is consistent with those applied in previous studies, subject to available data. In particular, three models were developed as shown in Fig. 1. With the use of these three complementary models, we seek to obtain insightful information regarding access to health care, utilization of primary health care resources and achievement of health outcomes.

Model 1 aims to assess the equitable distribution of human resources across CCGs, based on the registered patients list, by age group. In doing so, we aim to capture horizontal equity ensuring that to equal needs correspond equal amounts of resources available and, hopefully, equal treatment. As shown in Fig. 1, the chosen input variables are number of patients, stratified by age. The stratification used follows that applied by the NHS Digital in the Health Survey for England 2019. In this model, age is used as a measure for health care need by the population.

In turn, the output variables in Model 1 are the Full-Time Equivalent (FTE) of each professional group to inform on the human resources available in each CCG. In addition to the General Practitioners (GPs) and Nurses, two other staff categories are considered: All Direct Patient Care Staff and Administration and Non-clinical Staff. According to NHS Digital [39], Direct Patient Care Staff refers to Health Care Assistants, Care Coordinators, Dispensers, Phlebotomists, Pharmacists, Podiatrists, Physiotherapists, Physician Associates, Apprentices, Paramedics, Nursing Associates, Trainee Nursing Associates, Social Prescribing Link Workers, Improving Access to Psychological Therapies (IAPT) staff, Trainee IAPT Staff, Health Support Worker, and Other Direct Patient Care. Whereas Administration and Non-clinical Staff refers to Managers, Management Partners, Medical Secretaries, Receptionists, Telephonists, Estates and Ancillary, Apprentices, and Other Admin/non-clinical staff. The inclusion of all these professions intends to realistically demonstrate the functioning of primary health care services, following the recommendation of Neri et al. [5].

Model 2 aims to measure service efficiency, analysing the quantity of services delivered versus the resources used to deliver them. In health care, efficiency is of central importance as available resources are limited, therefore an efficient health system is one in which resources are optimally converted into population gains [40].

As shown in Fig. 1, the chosen input variables are the human resources allocated to each CCG, measured by the number of FTE members of staff in each professional group as described above. As output variables, activity indicators are used. In particular, we use the count of appointments by mode, which cover three main categories – face-to-face, home visits and others. The variable “Count of appointments by mode: other” reflects appointments that are held via telephone, video or online and those that are classified as unknown mode.

Model 3 aims to analyse if the services delivered produce desired outcomes. In specific, this model measures service effectiveness in reducing preventable emergency admissions and in maximizing patient satisfaction. As shown in Fig. 1, the chosen input variables are the number of emergency admissions for acute conditions that should not usually require hospital admission and the estimated number of patients who are not happy with the services received in the CCGs. The second input uses data from the GP Patient Survey, which provides an insight regarding the level of patient satisfaction towards the primary health care services provided. The use of patients’ level of satisfaction is important to track service quality [25]. On the output side, we have the number of patients stratified by age, reflecting the characteristics of the patients served.

Considering that it is important to identify determinants of poor performance, model 2 (efficiency) was run using both a CRS and a VRS assumption. Using these assumptions, we can identify the CCGs that were operating at an ideal size and the ones that displayed increasing or decreasing returns to scale. This kind of information is highly valuable for managers and policy makers as a major restructuring has taken place in the sector with the merging of several CCGs into larger ICSs.

On the contrary, model 1 and model 3 were run only with a CRS assumption considering that it is reasonable to assume that variations in the inputs should be associated with proportional variations in the outputs. Specifically, in model 1 it is reasonable to assume that variations in the number of patients registered should lead to proportional variations in the volume of resources allocated. Similarly, in model 3, it is reasonable to expect that variations in the number of patients registered should be associated with proportional variations in the volume of emergency admissions and in the number of unsatisfied patients.

An output orientation was used in Models 1 and 2, as we adopted the view shared by several researchers that in health care the focus should concentrate on increasing outputs instead of reducing inputs and costs. Furthermore, an output orientation is particularly relevant when there is evidence of waiting lists for services, as it is the case in England, the focus of our research. In the case of Model 3, considering that the CCGs only had control over the inputs, an input-oriented model was chosen. The rationale behind this model is to evaluate, for CCGs with comparable needs, which ones delivered a more effective service (i.e., which ones minimized preventable emergency admissions and the number of patients unsatisfied with the services received). The choices regarding model orientation reflect real-world challenges in primary health care delivery. Regarding equity assessment, the challenge is to secure the highest level of resources for each CCG to meet the needs of the population it serves. In terms of efficiency assessment, CCGs face the challenge of maximizsing the volume of services delivered within the available resources. Finally, in terms of effectiveness, the challenge is to minimize undesirable outcomes (such as emergency admissions and patient dissatisfaction), considering the volume and age structure of enrolled patients. Alternative orientations would not be suitable for our models, as they would generate improvement targets that would not be relevant to the CCGs.

For each model, weight restrictions were applied following the production trade-offs approach proposed by Podinovski [41], aiming to produce more meaningful results. Details of the weight restrictions used are presented in the Appendix.

The super-efficiency procedure proposed by Banker and Chang [42] for outlier identification was also used. However, considering that no CCG obtained a score higher than 140% in any of the three models (with only three CCGs marginally exceeding 130%), we decided not to exclude any CCGs from the analysis.

In turn, the MI proposed by Camanho and Dyson [11] was used to measure group performance when accounting for the level of deprivation. This method aims to assess the performance of two groups of CCGs, one including the CCGs presenting the highest levels of deprivation and the other one including the CCGs presenting the lowest levels of deprivation, as measured by the IMD scores. These two groups were formed according to the median value. The first group (A) has a value of IMD that is higher than or equal to the median, indicating greater deprivation. The second group (B) has a value of IMD that is lower than the median, indicating lower deprivation. Using these two groups, the three models were run to calculate performance spread, frontier gap and overall performance gap, generating valuable insights on how the performance of the CCGs was influenced by deprivation and how to best address health inequalities.

### Equity, efficiency and service effectiveness data and results

In this study, the performance and inequalities in primary health care in England were assessed using CCGs as DMUs, based on data from the NHS digital website for the calendar year 2019. Out of 191 CCGs, 3 were excluded due to incomplete activity data, resulting in an analysis of 188 CCGs. The exclusion of these three CCGs is not problematic to the representativeness of our results considering that a sample of 188 CCGs is sufficiently large to allow us to identify performance improvement. Furthermore, we have no reason to believe that the excluded CCGs have different characteristics to those included in the analysis. The choice of the 2019 calendar year was based on data availability and on the intention to avoid using data that could have been affected by the COVID pandemic.

Having previously justified the choice of variables and assumptions regarding each model, the results will now be presented and discussed.

#### Equity results

The results of Model 1 reveal that 11 out of the 188 CCGs analysed had an equitable distribution of human resources considering their populations’ needs. These were: NHS Bradford City CCG, NHS Camden CCG, NHS City and Hackney CCG, NHS Corby CCG, NHS Hambleton, Richmondshire and Whitby CCG, NHS Islington CCG, NHS Liverpool CCG, NHS North Cumbria CCG, NHS North Norfolk CCG, NHS Rushcliffe CCG and NHS Tower Hamlets CCG.

**Fig. 2:**
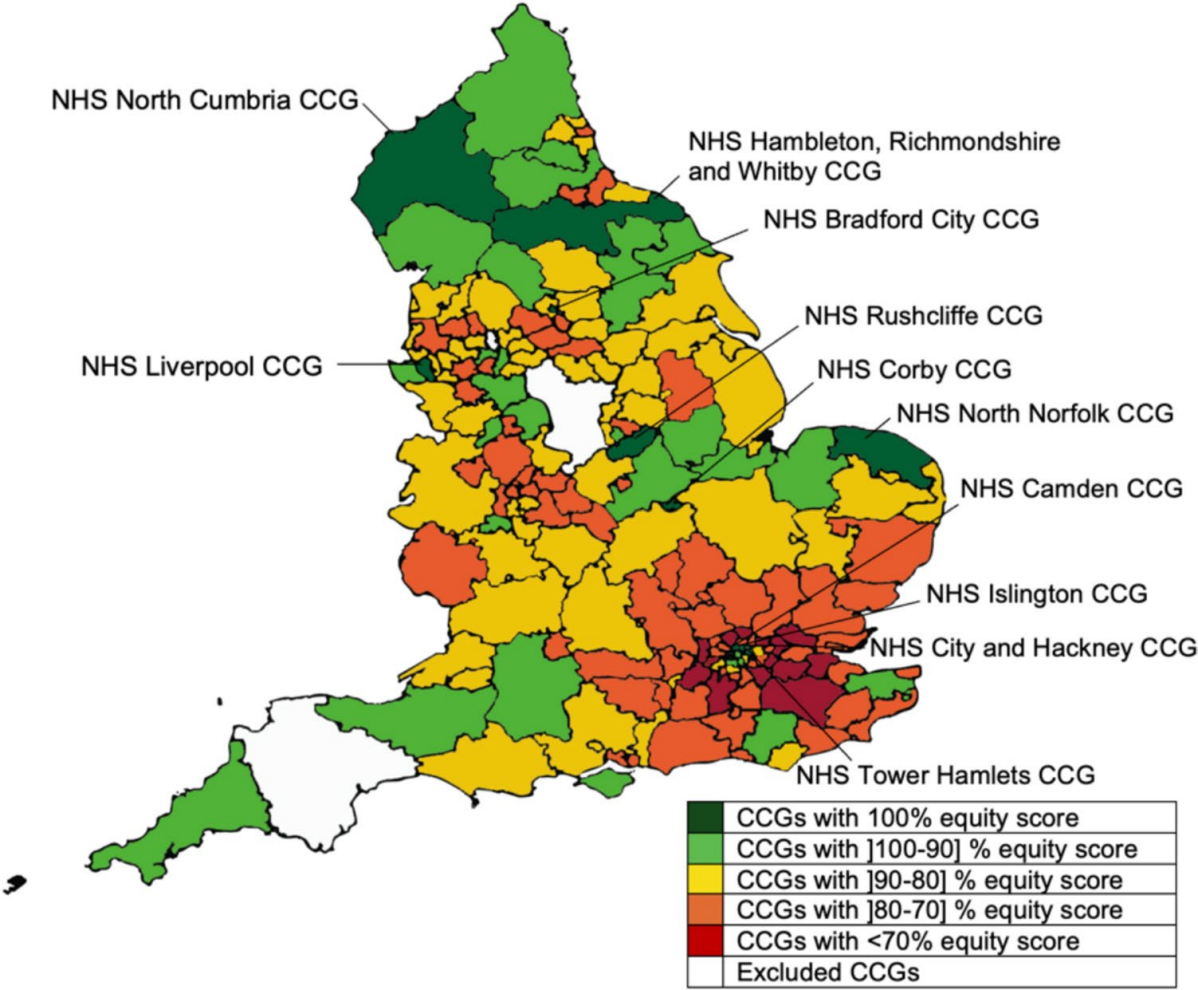
Map of England representing equity results obtained with DEA for Model 1

The average relative resource equity rate was 82.16%, with a standard deviation of 9.38%, suggesting considerable potential for improvement. Of the 177 CCGs with relative lower levels of performance, 14 had a relative resource equity rate below 70%, suggesting that comparatively to others, they did not seem to have enough resources to address the health needs of the population served. The CCG with the lowest relative resource equity rate was NHS Redbridge CCG with a score of 61.68%. If we compare the number of patients in this CCG per FTE GP or FTE Nurse, comparatively to the numbers in its peers (i.e., NHS Rushcliffe CCG, NHS Camden CCG, NHS Corby CCG), it becomes clear that the number of registered patients in all age groups in this CCG per FTE GP was higher than in its peers. The only exception is in the patient groups between 16–24 where the ratio in the NHS Camden CCG was marginally higher, and in the age group between 65–74 where the ratio in the NHS Rushcliffe CCG was also slightly higher. A similar conclusion can be drawn if we look at the number of patients per FTE Nurses and FTE All Direct Patient Staff. Interestingly, however, some of the peers of the NHS Redbridge CCG presented higher patient to FTE Administrative and Non-clinical staff ratios in several of the patients’ age groups. The results of Model 1 are illustrated in Fig. 2.

When it comes to identifying the best CCG practices in terms of Equity, we highlight the NHS Corby CCG, the NHS North Norfolk CCG and the NHS Rushcliffe CCG, which could act as benchmarks to 171, 144 and 100 of the poor performer CCGs, respectively.

Interestingly, the results also seem to suggest that CCGs operating in the most deprived areas presented higher scores in terms of the measure of human resource distribution equity based on the list of registered patients, by age group. If we consider the median of the IMD as the clustering point, we observe that those CCGs operating in areas with lower deprivation levels obtained an average score of 80.59% and 4 CCGs in this group belonged to the equity frontier. In turn, the CCGs operating in the most deprived areas presented an average score of 83.74%, and 7 CCGs in this group belonged to the equity frontier. Nevertheless, these results are inconclusive regarding whether the equity frontier of either of these two groups dominates the other. The findings of this analysis will be discussed in Sect. 3.3.

It is also interesting to observe that some important differences exist in the weight distribution of the variables in Model 1, especially at the output level, between the least deprived and the most deprived CCGs. For example, on average, the least deprived CCGs allocated a virtual weight 13% higher to the variable FTE GP than the one allocated by the CCGs operating in the most deprived areas. Contrarily, when it comes to the variable FTE Administrative and Non-Clinical staff, the average weight allocated to this variable by the most deprived CCGs was 11.1% higher than that allocated by the least deprived CCGs. This indicates that although more resources were allocated to the most deprived CCGs, these were not necessarily clinical staff. Although at the levels of the inputs some differences were also observed, these were minimal.

#### Efficiency results

The results of Model 2 reveal, in turn, that 12 CCGs were technically efficient: NHS Ashford CCG, NHS Bradford City CCG, NHS Calderdale CCG, NHS Cannock Chase CCG, NHS Hastings and Rother CCG, NHS Hounslow CCG, NHS Nottingham West CCG, NHS North East Essex CCG, NHS North Kirklees CCG, NHS Swale CCG, NHS Thanet CCG and NHS Wyre Forest CCG.

**Fig. 3:**
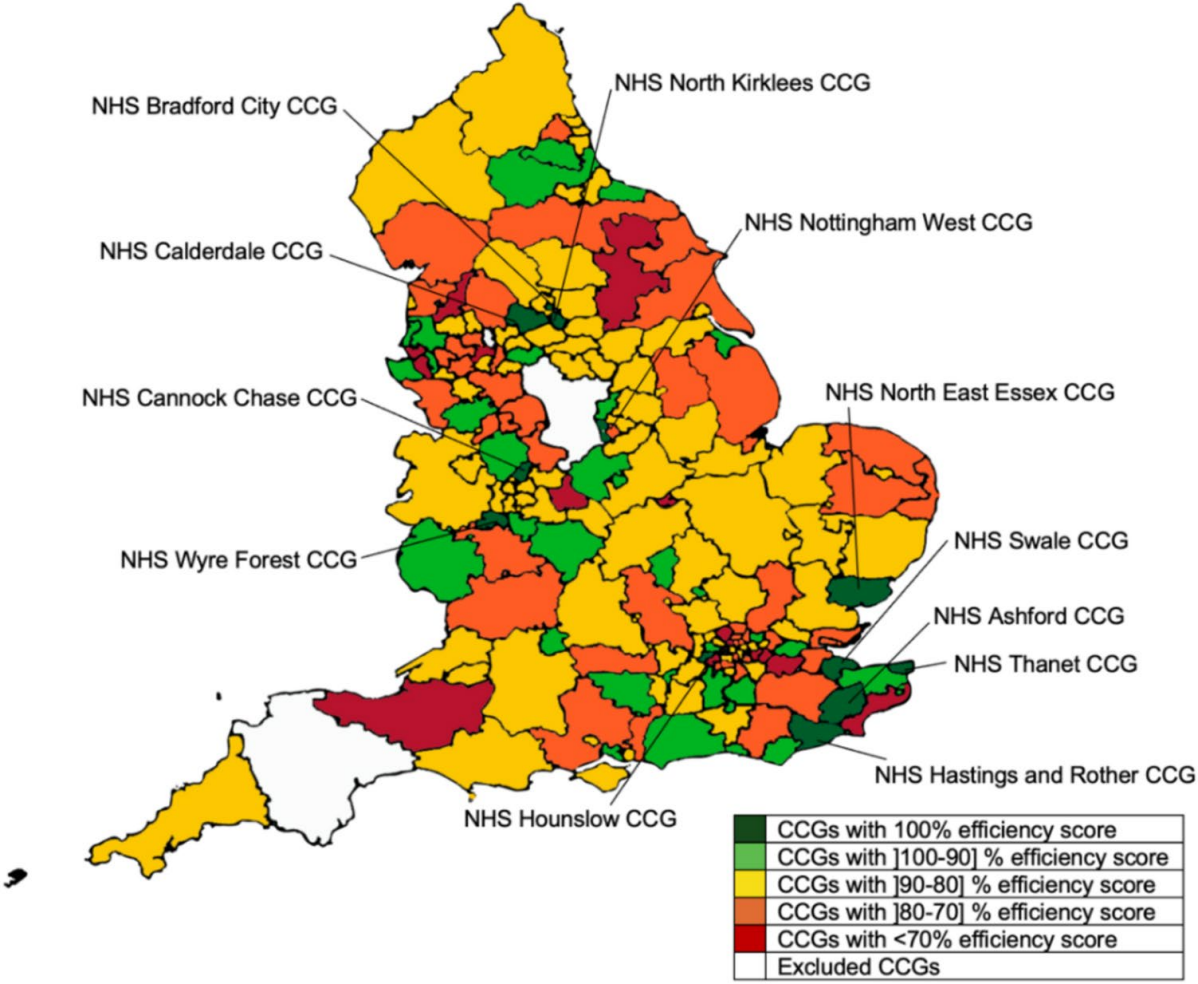
Map of England representing technical efficiency results obtained with DEA for Model 2

The average technical efficiency score of the 188 CCGs was 83.25% suggesting considerable room for enhancement, not only because the overall average score was well under 100%, but also because 15 CCGs presented efficiency scores below 70%. Of the inefficient units, NHS Dartford, Gravesham and Swanley CCG, NHS Richmond CCG and NHS Bexley CCG obtained the lowest scores (28.80%, 51.51% and 52.03%, respectively) and, consequently, presented the most potential for improvement. The technical efficiency results are illustrated in Fig. 3.

Scale inefficiency did not seem to be a major issue as the average scale efficiency score of the 188 CCGs was 95.89% and no CCG presented a scale efficiency score below 80%. Despite this, the analysis of the pure technical efficiency and scale efficiency scores allows us to draw very interesting conclusions. Firstly, the results show that there were 16 CCGs that were not technically efficient but were pure technically efficient, meaning that their inefficiency derived exclusively from scale. Secondly, the results show that only 53 out of the 188 CCGs analysed were operating at an optimal or close to optimal scale (i.e., scale efficiency scores equal or higher than 99%). Thirdly, the results show that from the 24 CCGs that presented larger scale problems (i.e., a scale efficiency score below 90%), only the NHS Darlington CCG was operating under increasing returns to scale (IRS), with the other 23 CCGs operating under decreasing returns to scale (DRS). This suggests, therefore, that most of the CCGs with evidence of scale problems were already operating at a larger than optimal size. This finding is relevant because in recent years several mergers have taken place in the sector. For example, of the units that were operating under decreasing returns to scale, NHS Leeds CCG, NHS Birmingham and Solihull CCG, NHS Bristol, North Somerset and South Gloucestershire CCG, NHS Buckinghamshire CCG, NHS Berkshire West CCG, and NHS East Berkshire CCG, were CCGs that were created in 2018 resulting from mergers of other CCGs. These results seem to support the conclusion that these mergers had a detrimental impact on the scale efficiency of these CCGs. Considering that ICSs, formally introduced in 2022, cover the same or larger catchment areas than those of the CCGs, scale inefficiency may pose a challenge for these newly created entities. For example, the ICS Norfolk and Waveney covers exactly the same area that was covered by the NHS Norfolk and Waveney CCG, a unit that resulted from merging 5 CCGs all operating under DRS. This suggests that the resulting unit may also face DRS due to its very large size. Several other examples of ICSs that resulted from mergers of CCGs already operating under DRS can be found (e.g., Lincolnshire ICS, Surrey Heartlands ICS, North Central London ICS, South East London ICS). Considering this result, it is recommended that ICSs regularly evaluate their scale efficiency. For ICSs found to be operating under decreasing returns to scale, creating several intermediate management tiers may be beneficial. This could involve forming smaller, geographically based local groups with autonomous management. Such a structure would address scale efficiency issues without compromising care integration.

Among the benchmarks in terms of technical efficiency, we highlight the NHS Hounslow CCG, the NHS Nottingham West CCG and the NHS North Kirklees CCG, which acted as references for 131, 118 and 95 of the poorer performer CCGs, respectively. For example, NHS Bexley CCG showed significant potential for improvement in terms of efficiency, with NHS Calderdale CCG and NHS North Hampshire CCG serving as benchmarks. Meetings could have been arranged with professionals from these two CCGs to learn about their structures and processes. Following these meetings, an improvement plan could have been developed for NHS Bexley CCG.

The results of Model 2 also seem to suggest that those CCGs operating in the most deprived areas presented higher efficiency scores than those operating in least deprived areas, although the difference was not significant. The former presented an average technical efficiency score of 83.97%, with 9 CCGs forming the efficiency frontier. The latter had an average efficiency score of 82.53% and 3 CCGs were in the efficiency frontier.

Similarly to Model 1, it is also interesting to observe some important differences in the weight distribution between the least deprived and the most deprived CCGs, which confirm some of the conclusions drawn previously. For example, at the level of the resources, the weight allocated to Administrative and Non clinical staff was higher in the least deprived CCGs than in the most deprived, reinforcing the fact that the latter had higher proportions of this category of staff than the former. The opposite situation was observed when it comes to the weights allocated to the GPs. At the level of the outputs, no significant differences were observed.

#### Effectiveness results

**Fig. 4:**
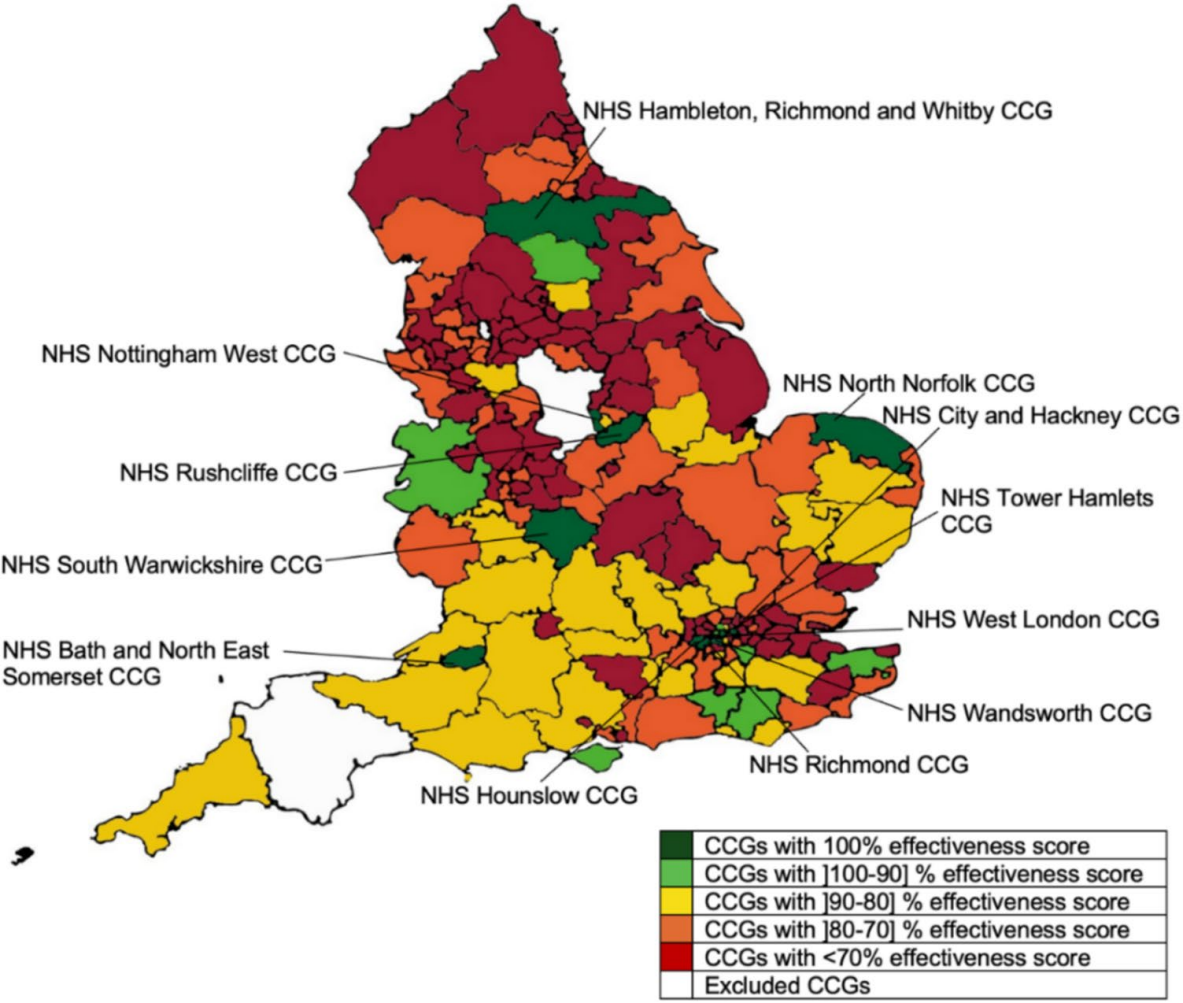
Map of England representing service effectiveness results obtained with DEA for Model 3

Finally, the results of Model 3 reveal that 12 CCGs were effective: NHS Hounslow CCG, NHS South Warwickshire CCG, NHS Wandsworth CCG, NHS Tower Hamlets CCG, NHS West London CCG, NHS Bath and North East Somerset CCG, NHS City and Hackney CCG, NHS Rushcliffe CCG, NHS North Norfolk CCG, NHS Hambleton, Richmondshire and Whitby CCG, NHS Nottingham West CCG and NHS Richmond CCG. These were the CCGs that obtained the best results in terms of preventable emergency admissions and patient satisfaction, considering the population served, stratified by age groups. From the three performance dimensions analysed, this is clearly the one where larger asymmetries between the performance of the CCGs were observed. The overall effectiveness score was 73.25%, with 86 CCGs presenting a score under 70%. Amongst the least effective we highlight the NHS Swindon CCG, NHS Bradford City CCG and NHS Luton CCG, which obtained effectiveness scores of 51.90%, 47.87% and 46.68%, respectively. The effectiveness results are illustrated in Fig. 4.

In terms of the benchmarks, the NHS Rushcliffe CCG, NHS West London CCG and NHS Bath and North East Somerset CCG were the ones that stood out as best models to improve the effectiveness of a large number of underperforming CCGs as these 3 CCGs were a benchmark for 125, 109 and 98 CCGs, respectively.

Interestingly, however, and contrarily to the findings of Models 1 and 2, when we compare the performance of the CCGs operating in the least deprived areas with the performance of the ones operating in the most deprived areas, we conclude that the former outperformed the later in around 10%. More specifically, the average effectiveness score of the CCGs operating in the least deprived areas was 78.11%, while the average score of the ones operating in the most deprived areas was 68.39%. The number of CCGs of the first group in the frontier was also twice the number of the CCGs defining the frontier in the second group (8 against 4). While these findings are interesting, similar to what was mentioned previously, they do not allow us to conclude whether the two groups have different frontiers. This analysis is carried out in the next section.

In terms of the weight structure of the different variables, no major differences can be observed between these two groups at the level of the inputs, with the average virtual weight being uniformly distributed between the two variables. When it comes to the outputs, most of the output weights was allocated to the patient groups aged 0 to 15 and 75 or more, with these two variables accounting, on average, with 69.2% of the virtual weight in the case of the least deprived CCGs, and with 59.6% in the case of the most deprived CCGs. These results are explained, in part, by the weight restrictions imposed on Model 3.

### Malmquist Index data and results

The analysis in the previous sections reveals that important differences existed in the performance of the CCGs operating in areas with different deprivation levels. In particular, it shows that CCGs in most deprived areas seemed to outperform, on average, those in least deprived areas in terms of equity and efficiency, but the opposite was observed in terms of effectiveness. However, the results obtained so far do not allow us to conclude whether the performance frontiers of either of these two groups dominated the other. In order to clarify this issue, we used the approach discussed in Sect. 2.3.

The common factor used to group DMUs into two groups was the Index of Multiple Deprivation average scores. After dividing the 188 CCGs into two groups – first group (A): higher IMD score (94 CCGs) and second group (B): lower IMD score (94 CCGs), the three models described previously where run using the EMS Software [43] with a constant returns to scale approach as suggested by Camanho and Dyson [11]. In consistency with the analysis presented before, an output orientation was used for Models 1 and 2 and an input orientation was used for Model 3. The same weight restrictions described previously for each model were applied. The results obtained allowed us to calculate the values of performance spread (${IE}^{AB}$), frontier shift (${IF}^{AB}$), and overall performance (${I}^{AB}$) for each model.

For the three models, the aim was to assess if deprivation affects equity, efficiency and effectiveness levels. The results of this analysis are presented in Table 1.

**Table 1:** Results obtained for the three DEA models using the MI by Camanho and Dyson [11]

Malmquist Indices	*Model 1* *Human resource distribution equity based on registered patients list, by age group*	Model 2 *Service efficiency*	Model 3 *Service effectiveness*
Efficiency Spread ($${IE}^{AB})$$	0.991	1.003	1.014
Frontier Shift (${IF}^{AB}$)	1.072	1.024	0.871
Overall Performance (${I}^{AB}$)	1.063	1.027	0.883

For Model 1, the result of the equity spread is 0.991, which implies that equity scores were more consistent (less dispersed) in the less deprived CCGs compared to the most deprived group. In other words, in the less deprived areas, the average resource equity level observed was higher than the average observed in most deprived areas. However, the results also show that the frontier shift is higher than one, which means that there was dominance of the best practice frontier in the most deprived areas. More specifically, the frontier of access to resources of the most deprived CCGs was 7.2% higher than the least deprived CCGs frontier, considering the level of needs. These results show that although the frontier of the CCGs operating in the most deprived areas dominated, on average, the one of the CCGs operating in the least deprived areas, the former CCGs were, on average, further away from the respective frontier than the least deprived. When the two effects are put together, we observe that the overall equity performance is 1.063, which means that, on average, the available human resources, when accounting for needs, were 6.3% higher in the most deprived areas than in the least deprived areas. These results emphasize a positive discrimination, where there is an overcompensation of human resources in the most deprived areas in detriment of the least deprived areas. This is consistent with the findings discussed in Sect. 3.2.1.

Also, in Table 1, we can see that both the efficiency spread and the frontier shift of Model 2 is higher than one (i.e., 1.003 and 1.024, respectively). These results reflect marginally higher service efficiency levels, and consequently lower dispersion of efficiency in the most deprived CCGs. They also reflect that the best practice frontier of the most deprived CCGs was, on average, at a 2.4% higher level than that of the least deprived CCGs. The overall performance in Model 2 is 1.027, which indicates that, on average, service efficiency, considering the number of appointments, was 2.7% higher in the most deprived areas than the level observed in the least deprived areas.

In Model 3, the effectiveness spread is 1.014, which, once again, reflects higher service effectiveness level consistency (lower dispersion) in the most deprived CCGs. However, the value of the frontier shift is 0.871, which indicates that the frontier of the most deprived CCGs was at a much lower level than that observed for the least deprived areas. This reflects that service effectiveness levels in most deprived CCGs were considerably lower than in the least deprived CCGs. Finally, the overall performance for the third model is 0.883, which means that, on average, service effectiveness in reducing emergency admissions and patients’ unsatisfaction was considerably lower in the most deprived CCGs than in the least deprived CCGs.

These results have important implications because they indicate that the health and social policies in place in England to reduce health inequalities caused by deprivation are having a positive impact in terms of equity and efficiency, but further efforts need to be carried out to correct the still existing asymmetries regarding service effectiveness. Future research should examine not only the determinants of the still prevalent asymmetries but also the dynamic evolution of the different components of the MI to further assess the impact of the recent reforms undertaken in the PHC system.

### The practical and policy implications of the results

Despite the exploratory nature of this study, there are important considerations that can be derived from the results previously presented. These results can be divided into two parts: performance measurement of primary health care in the NHS using DEA and measurement of the effect of deprivation on primary health care performance using the MI.

The performance results obtained through DEA assess equity, service efficiency and service effectiveness. The equity results identify that only 11 of the 188 CCGs stood apart from the others as having the most human resources to address the needs of the registered patients list, by age group. Improvement was needed to reach the equity of human resources among the other CCGs. Although CCG budget allocation was calculated based on health needs of that population (of which age is one criteria), having an adequate budget may not equate to sufficient health professionals. In England, there is a significant shortage of health care staff, for which new governmental strategies and increased effort are needed to recruit and retain skilled health professionals. Another option would be to relocate some health professionals to reach a more equitable distribution of resources. In this respect, it is important to regularly assess the equitable distribution of resources across ICSs to identify areas that lack sufficient resources. For example, ICSs in the South East region should be closely monitored to enhance resource allocation, as our study reveals strong disparities in terms of equity.

Our results also highlight inequities in workforce distribution, demonstrating that while some CCGs had a disproportionately high number of health care professionals, others faced staffing shortages. Although budget allocations are determined based on population health needs, the actual distribution of health care professionals remains inequitable.

This information can be used to optimize workforce planning strategies, ensuring that budget allocation for ICSs translates into equitable access to health care professionals for the patient populations they serve. Implementing robust recruitment and retention policies, alongside increased funding, may enhance equitable access to health care professionals. Additionally, it is crucial to assess whether ICSs are receiving the appropriate type of resources, for example, prioritising clinical staff over non-clinical and administrative personnel, where necessary.

Service efficiency results based on staff and services produced, identified that 12 CCGs were technically efficient, pure technically efficient and scale efficient. However, 62 CCGs had technical efficiency levels under 80% and 39 had scores of pure technical efficiency under 80%, which required improvement. NHS Hounslow CCG, NHS Nottingham West CCG and NHS North Kirklees CCG, served as benchmarks to a high number of inefficient units. A practical way to improve efficiency levels of the inefficient units would be to promote learning networks and discussion sessions between them and their respective benchmarks. In these sessions, work methods, staffing and roles, management and policies should be discussed, for inefficient units to learn from and follow the efficient units. It is important to emphasize that primary health care is part of the NHS, which was founded to provide equal care to equal needs. For this to happen, the system should work united to reach this goal. Even though CCGs, and now the ICSs, divide England in areas, they are all part of a public service, for which there should be a joint effort to improve the results of the areas that are not reaching their optimal efficiency. The more efficient the health system is as a total, the better value for money the taxpayer will receive.

The results of the analysis also provide valuable information for policymakers and managers regarding scale efficiency. As previously discussed, the newly formed ICSs are large entities, some of which correspond to the merger of several CCGs already operating under DRS. This suggests that challenges may be faced by ICSs regarding scale efficiency. Further analyses with more recent data should be conducted to evaluate this aspect.

Regarding service effectiveness, only 12 CCGs were identified as being effective in reducing avoidable emergency admissions and in maximizing patient satisfaction, which demands attention as primary health care is the first point of access to the health system. If avoidable emergency admissions are not reduced through effective primary health care, more patients will require secondary and tertiary care, which ultimately is more costly to the taxpayer and may cause delayed treatment, worsening health outcomes for the population. Effective primary health care should include health monitoring and disease screening throughout the life cycle, inoculations, prenatal care, chronic illness management, and more. If these basic activities are not being carried out in an effective manner, this will lead to emergency admissions to hospital. Simultaneously, promoting a positive patient experience is essential for the patients to access and utilize primary health care services as a first choice and to reach the desired health outcomes. To improve service effectiveness, governmental action is required to create better health pathways and encourage primary health care utilization. At a more local level, ineffective organizations may benefit from learning from the effective ones, as mentioned previously. The effective CCGs that served as a benchmark to the highest number of ineffective units were NHS Rushcliffe CCG, NHS West London CCG and NHS Bath and North East Somerset CCG.

The results obtained through the MI provide valuable information on the effect of deprivation on primary health care performance. The results for the first and second model show that there was a positive discrimination in the allocation of resources favoring the CCGs that operated in the most deprived areas, and that these CCGs were slightly more efficient in the use of the resources than the CCGs operating in the least deprived areas. In both models the best practice frontier of the CCGs operating in the most deprived areas dominated the frontier of the CCGs operating in the least deprived areas. On the other hand, the third model indicates that despite the CCGs in more deprived areas having more resources and being more efficient, these achievements were not enough to compensate for the increased health needs of the populations they served as these CCGs were considerably less effective in preventing emergency admissions and in increasing patient satisfaction than the CCGs in least deprived areas. The IMD was used in the formula to calculate CCGs budgets, and our results seem to confirm that this way of allocating resources was reaching its objective in terms of equity and efficiency. However, more needs to be done to correct the still existing asymmetries in terms of effectiveness. It is important to bear in mind that our results indicate that although more resources seemed to be allocated to CCGs in more deprived areas, these extra resources predominantly consisted of non-clinical staff, which might explain, at least partially, why despite having more resources, these CCGs were outperformed by those operating in least deprived areas.

One of the objectives of establishing ICSs was to reduce health inequalities. Our results suggest that improving health outcomes, particularly, in most deprived areas, should indeed be a priority for ICSs managers. The CCGs identified as most effective could be important sources of information regarding how to improve health outcomes. For example, South East London ICS is formed by several former CCGs, including Greenwich CCG. Despite facing high deprivation, this CCG achieved an effectiveness score of 100% and serves as a benchmark to 84 CCGs. The structures and processes used by Greenwich care providers can serve as inspiration for South East London ICS managers.

Another important implication of this research is that it shows that the two techniques used are valuable tools to assess and improve performance in primary health care. For example, using the two techniques, health care managers and policy makers can identify which of the newly created ICSs are the most successful in transforming limited resources into precious health outcomes. Not only will they be able to identify which ICSs are most successful and which are least successful, these techniques also allow them to understand from which successful units it is more beneficial to learn from. Although these techniques provide results from the past, they can contribute to improve the future.

Despite covering only the calendar year of 2019 and focusing on CCGs, as previously detailed, the results of our study provide valuable information for the new ICSs and policymaking. In particular, this information can contribute to improving access, utilization, and outcomes of primary health care in the NHS.

To translate our findings into actionable strategies, ICS managers could implement several immediate steps.

Firstly, to address the inequitable distribution of health care professionals, workforce audits should be conducted to assess whether resources are being allocated proportionally and appropriately based on health care needs. Additionally, ICS managers operating in understaffed areas should develop targeted recruitment and retention programmes to attract health care professionals to underserved regions. Exploring incentives, such as financial benefits, career development pathways, and remote/hybrid working opportunities can further enhance workforce recruitment and retention.

Secondly, to improve service efficiency, ICS managers should organize efficiency-focused peer-learning sessions between underperforming ICSs and their benchmark counterparts. These sessions should facilitate discussions on staffing, work methods, autonomy levels, digital tool implementation, patient flow, and patient management. For example, understanding differences in professional roles and autonomy levels, such as whether Nurse Practitioners should have non-medical prescribing rights, or the scope of practice for Physician Associates, may inform potential optimization strategies that could be implemented in lower-performing ICSs.

Thirdly, our results indicate that several CCGs that merged to form ICSs were already operating under Decreasing Returns to Scale before the merger. This suggests that ICS managers should conduct post-merger efficiency assessments to determine whether these structural changes have led to the desired performance improvements or if further restructuring is necessary. As demonstrated in our study, the DEA technique would be an appropriate methodological approach for conducting such assessments.

Fourthly, the results of our study on service effectiveness, particularly in relation to avoidable emergency admissions, underscore the need for enhanced preventative health care within the NHS. ICS managers should implement initiatives aimed at increasing patient engagement with primary health care services, thereby reducing reliance on hospital services. Potential strategies include increasing the number of available daily appointments, reducing the gap between booking and appointment dates, and expanding out-of-hours primary care services. Additionally, health education programmes should be developed to empower patients with chronic conditions to manage their health effectively. These programmes should focus on identifying early warning signs of deterioration and guiding patients on when to seek primary care intervention, thereby reducing emergency hospital admissions.

Lastly, ICS managers should strive to enhance patient experience and trust in primary health care services by improving accessibility through the strategies mentioned above, enhancing communication channels, particularly for individuals with sensory impairments, and implementing culturally sensitive health care approaches. This can be achieved by providing mandatory training for health care professionals on cultural inclusion and awareness. After implementing these measures, ICS managers should continue to utilize the GP Patient Survey to assess overall patient satisfaction and identify areas for further improvement.

By implementing these data-driven, evidence-based interventions, ICS managers can ensure that their strategic objectives align with broader NHS policy goals. These goals include reducing health inequalities, enhancing the equitable allocation of resources, improving service efficiency and effectiveness, maximizing taxpayer value for money, ensuring timely access to appropriate care, and enhancing overall patient satisfaction with primary health care in England.

## Conclusion

Health inequalities have been identified as undesirable and detrimental to the health of a population, which has led NHS England to recognize them as a central concern and to concentrate efforts to reducing them. The results of this study demonstrate that the policies addressing deprivation that were in place in 2019 were having a positive effect on primary health care performance in terms of equity and efficiency, but that they were still not enough to address the asymmetries in terms of the effectiveness of the services provided.

Through the literature review it was possible to conclude that DEA is a commonly used technique to assess performance in health care systems and more specifically in primary health care, however, to the best of our knowledge, there are no published studies that use the MI to contrast the performance of PHC providers according to the level of deprivation. Therefore, the application documented in this study is innovative and has the potential to inform not only theory but also to assist policymakers and managers.

Despite the exploratory nature of this research, there are some relevant empirical findings from this study. Initially three DEA models were run to assess resource allocation equity, service efficiency and service effectiveness among CCGs. All the models produced relevant results identifying the units that had best performance and those with the lowest. Simultaneously, this technique allows the identification of learning peers (benchmarks), which provides useful information regarding the best practices to learn from to improve the overall performance of PHC providers in the NHS. By identifying the lowest performing units, it creates an opportunity to investigate the causes of this poor performance and to compare their practices against better performing units. It is important to remember that health service providers within the NHS should work together to reach the best health outcomes for the national population, therefore a collaborative effort should be applied to improve the performance of all the primary health care system. This is one of the most important aims of the newly established ICSs.

Scale efficiency was also analysed not only to identify those CCGs that were operating with an optimal size, but also to explore the likely success of the large ICSs created in 2022. This analysis shows that some of the mergers that took place between CCGs might have exacerbated some scale issues and provides a valuable framework to inform future NHS reorganizations.

Of the three aspects of performance (equity, efficiency and effectiveness) measured through DEA in this study, service efficiency was the aspect that presented best performance results among the CCGs studied, albeit all three aspects requiring significant improvement. The results obtained are also indicative that some kind of trade-offs may take place between these three performance dimensions, meaning that it might be difficult to excel in the three simultaneously.

The second technique applied – the Malmquist Index, allowed us to contrast the performance of the group with highest level of deprivation with the group with lowest level of deprivation. This method provided very interesting results, confirming that the health and social policies addressing deprivation that were in place in 2019 seemed to have a positive effect on performance in terms of equity and efficiency. However, important asymmetries were still observed in terms of service effectiveness, suggesting that further measures need to be adopted to complement the existing ones.

In fact, our study’s findings demonstrate that deprivation significantly impacts primary health care performance. While CCGs operating in the most deprived areas received favourable resource allocation, their service effectiveness- measured by their ability to reduce avoidable emergency admissions and improve patient satisfaction- remained lower than that of CCGs in less deprived areas. These findings align with the NHS’s focus on Core20PLUS5 (a key initiative under the National Healthcare Inequalities Improvement Programme), suggesting that targeted interventions beyond mere resource allocation are necessary to enhance service effectiveness in deprived areas, thereby improving population health outcomes.

One of the main limitations to this research was data availability. Despite this, the research presented in this paper, demonstrates an innovative and useful method for measuring performance and the effect of deprivation on that performance. It is clearly a reproducible method that can be applied to other datasets from different calendar years and even to other common factors for distinguishing group performance (besides deprivation).

Creating valid weight restrictions was also a challenging step of this research, as not many authors provide insights on how to formulate meaningful weight restrictions.

As suggestions for further research, we see considerable potential in running these analyses again with more recent data. Since the data used in this study was collected, the long-term plan has been in place for five years (since 2019), and in July 2022, CCGs were dissolved and ICSs were created. It would be highly beneficial to assess the impact of these changes in policy and structure on primary health care in the NHS. Furthermore, if data become available, it would be relevant to include in the analysis, information on the size of the premises, the volume of diagnostic equipment available in each group, as well as information about improvements in health-related quality of life.

The interpretation of the results obtained in this study should be approached with caution, considering the data limitations discussed above. In this respect, if strong disparities exist in terms of the variables that were omitted from the analyses, the performance of some CCGs may have been misrepresented.

Although our study provides an important analysis and discussion on the likely impact of deprivation of the performance of the CCGs, the results report to a single year, and therefore, to have a more comprehensive assessment of the effectiveness of the health and social care policies in place, it would be desirable to compare not only the performance of groups of entities in a specific point in time, but also to compare their performance over time. This should also be the subject of further research.

Furthermore, we believe it would also be beneficial to interview policymakers and managers within primary health care in the NHS to obtain their feedback on the results of these studies and to understand how to incorporate them into their decisions and policies.

Even though our study focuses on NHS CCGs in England, the conceptual framework used can be applied to assess the performance of primary care providers in other countries, as it includes the most important indicators regarding inputs, outputs and outcomes of primary care delivery. The weight restrictions developed for our study can also be implemented to evaluate primary care providers in other countries, as they are based on production trade-offs that are not specific to England. Furthermore, the combination of DEA with the modified version of the Malmquist Index can also be applied to compare the performance of health care providers in other countries, depending on factors such as the level of deprivation or other relevant characteristics for analysis.

## Data Availability

Data will be made available upon request to the corresponding author.

## Appendix

### Weight restrictions applied to the three dea models

**Table:**

DEA Model 1- weight restrictions	DEA Model 2 – weight restrictions
WR1:${{\varvec{v}}}_{8}-{{\varvec{v}}}_{7}\ge 0$	WR1:${v}_{1}-{v}_{2}\ge 0$
WR2:${{\varvec{v}}}_{8}-{{\varvec{v}}}_{6}\ge 0$	WR2:${v}_{1}-{v}_{3}\ge 0$
WR3:${{\varvec{v}}}_{8}-{{\varvec{v}}}_{5}\ge 0$	WR3:${v}_{1}-{v}_{4}\ge 0$
WR4:${{\varvec{v}}}_{8}-{{\varvec{v}}}_{4}\ge 0$	WR4:${v}_{2}-{v}_{3}\ge 0$
WR5:${{\varvec{v}}}_{8}-{{\varvec{v}}}_{3}\ge 0$	WR5:${v}_{2}-{v}_{4}\ge 0$
WR6:${{\varvec{v}}}_{8}-{{\varvec{v}}}_{2}\ge 0$	WR6:${v}_{3}-{v}_{4}\ge 0$
WR7:${{\varvec{v}}}_{8}-{{\varvec{v}}}_{1}\ge 0$	WR7:${u}_{2}-{u}_{1}\ge 0$
WR8:${{\varvec{v}}}_{7}-{{\varvec{v}}}_{6}\ge 0$	WR8:${u}_{2}-{u}_{3}\ge 0$
WR9:${{\varvec{v}}}_{7}-{{\varvec{v}}}_{5}\ge 0$	WR9:${u}_{1}-{u}_{3}\ge 0$
WR10:${{\varvec{v}}}_{7}-{{\varvec{v}}}_{4}\ge 0$	
WR11:${{\varvec{v}}}_{7}-{{\varvec{v}}}_{3}\ge 0$	
WR12:${{\varvec{v}}}_{7}-{{\varvec{v}}}_{2}\ge 0$	**DEA Model 3 – weight restrictions**
WR13:${{\varvec{v}}}_{7}-{{\varvec{v}}}_{1}\ge 0$	WR1:${{\varvec{u}}}_{8}-{{\varvec{u}}}_{7}\ge 0$
WR14:${{\varvec{v}}}_{1}-{{\varvec{v}}}_{2}\ge 0$	WR2:${{\varvec{u}}}_{8}-{{\varvec{u}}}_{6}\ge 0$
WR15:${{\varvec{v}}}_{1}-{{\varvec{v}}}_{3}\ge 0$	WR3:${{\varvec{u}}}_{8}-{{\varvec{u}}}_{5}\ge 0$
WR16:${{\varvec{v}}}_{1}-{{\varvec{v}}}_{4}\ge 0$	WR4:${{\varvec{u}}}_{8}-{{\varvec{u}}}_{4}\ge 0$
WR17:${{\varvec{v}}}_{1}-{{\varvec{v}}}_{5}\ge 0$	WR5:${{\varvec{u}}}_{8}-{{\varvec{u}}}_{3}\ge 0$
WR18:${{\varvec{v}}}_{1}-{{\varvec{v}}}_{6}\ge 0$	WR6:${{\varvec{u}}}_{8}-{{\varvec{u}}}_{2}\ge 0$
WR19:${{\varvec{u}}}_{1}-{{\varvec{u}}}_{2}\ge 0$	WR7:${{\varvec{u}}}_{8}-{{\varvec{u}}}_{1}\ge 0$
WR20:${{\varvec{u}}}_{1}-{{\varvec{u}}}_{3}\ge 0$	WR8:${{\varvec{u}}}_{7}-{{\varvec{u}}}_{6}\ge 0$
WR21:${{\varvec{u}}}_{1}-{{\varvec{u}}}_{4}\ge 0$	WR9:${{\varvec{u}}}_{7}-{{\varvec{u}}}_{5}\ge 0$
WR22:${{\varvec{u}}}_{2}-{{\varvec{u}}}_{3}\ge 0$	WR10:${{\varvec{u}}}_{7}-{{\varvec{u}}}_{4}\ge 0$
WR23:${{\varvec{u}}}_{2}-{{\varvec{u}}}_{4}\ge 0$	WR11:${{\varvec{u}}}_{7}-{{\varvec{u}}}_{3}\ge 0$
WR24:${{\varvec{u}}}_{3}-{{\varvec{u}}}_{4}\ge 0$	WR12:${{\varvec{u}}}_{7}-{{\varvec{u}}}_{2}\ge 0$
	WR13:${{\varvec{u}}}_{7}-{{\varvec{u}}}_{1}\ge 0$
	WR14:${{\varvec{u}}}_{1}-{{\varvec{u}}}_{2}\ge 0$
	WR15:${{\varvec{u}}}_{1}-{{\varvec{u}}}_{3}\ge 0$
	WR16:${{\varvec{u}}}_{1}-{{\varvec{u}}}_{4}\ge 0$
	WR17:${{\varvec{u}}}_{1}-{{\varvec{u}}}_{5}\ge 0$
	WR18:${{\varvec{u}}}_{1}-{{\varvec{u}}}_{6}\ge 0$
